# Myrrh Inhibits LPS-Induced Inflammatory Response and Protects from Cecal Ligation and Puncture-Induced Sepsis

**DOI:** 10.1155/2012/278718

**Published:** 2011-08-02

**Authors:** Min-Sun Kim, Gi-Sang Bae, Kyoung-Chel Park, Bon Soon Koo, Byung-Jin Kim, Hye-Jin Lee, Sang-Wan Seo, Yong Kook Shin, Won-Seok Jung, Jung-Hee Cho, Youn-Chul Kim, Tae-Hyeon Kim, Ho-Joon Song, Sung-Joo Park

**Affiliations:** ^1^Department of Herbology, School of Oriental Medicine, Wonkwang University, Iksan, Jeonbuk 540-749, Republic of Korea; ^2^Department of Manufacturing Convergence Technology, Korean Institute of Industrial Technology, 1271-18, Sa-3 dong, Sangrok-gu, Ansan city, Gyeonggi-do 426-173, Republic of Korea; ^3^ChungBuk Technopark Bio Center, Jecheon, ChungBuk 390-250, Republic of Korea; ^4^Jeollanamdo Development Institute for Korean Traditional Medicine, Jangheung, Jeollanamdo 529-851, Republic of Korea; ^5^Standardized Material Bank for New Botanical Drugs, College of Pharmacy, Wonkwang University, Iksan 570-749, Republic of Korea; ^6^Department of Internal Medicine, College of Medicine, Wonkwang University, Iksan, Jeonbuk 570-749, Republic of Korea

## Abstract

Myrrh has been used as an antibacterial and anti-inflammatory agent. However, effect of myrrh on peritoneal macrophages and clinically relevant models of septic shock, such as cecal ligation and puncture (CLP), is not well understood. Here, we investigated the inhibitory effect and mechanism(s) of myrrh on inflammatory responses. Myrrh inhibited LPS-induced productions of inflammatory mediators such as nitric oxide, prostaglandin E_2_, and tumor necrosis factor-**α** but not of interleukin (IL)-1**β** and IL-6 in peritoneal macrophages. In addition, Myrrh inhibited LPS-induced activation of c-jun NH_2_-terminal kinase (JNK) but not of extracellular signal-regulated kinase (ERK), p38, and nuclear factor-**κ**B. Administration of Myrrh reduced the CLP-induced mortality and bacterial counts and inhibited inflammatory mediators. Furthermore, administration of Myrrh attenuated CLP-induced liver damages, which were mainly evidenced by decreased infiltration of leukocytes and aspartate aminotransferase/alanine aminotransferase level. Taken together, these results provide the evidence for the anti-inflammatory and antibacterial potential of Myrrh in sepsis.

## 1. Introduction

Inflammation plays key roles in many pathophysiological conditions [1], although it acts as an adaptive host defense mechanism against infection or injury. In inflammatory processes, macrophages are important players providing immediate defense against foreign agents. Upon activation, macrophages produce inflammatory mediators, such as tumor necrosis factor-*α* (TNF-*α*), interleukin (IL), leukotrienes, nitric oxide (NO), and prostaglandin E_2_ (PGE_2_) [2, 3]. Excess production of these cytokines and proinflammatory mediators is involved in many inflammatory diseases such as atherosclerosis, rheumatoid arthritis, asthma, pulmonary fibrosis, and septic shock [4]. Thus, inhibition of inflammatory responses is an important target in the treatment of inflammatory diseases.

3 major MAPKs have been defined: extracellular signal-regulated kinase (ERK), c-jun NH2-terminal kinase (JNK), and p38 [5, 6]. The activation of MAPKs is known to be required for the productions of NO and proinflammatory cytokines in activated macrophage cells [7]. MAPKs signaling pathways are involved in LPS-induced expression of iNOS, COX-2, and of proinflammatory cytokines in activated macrophages. Therefore, anti-inflammatory drug development could include, in addition to MAPKs inhibitors, agents that block overproduction of inflammatory mediators by inhibiting the expression of inflammatory mediators and proinflammatory cytokines.

Severe sepsis has a significant impact on public health, with an estimated incidence of nearly 800,000 cases per year, resulting in over 200,000 deaths at an annual cost of over $17 billion [8]. Sepsis develops when the initial host response fails to contain the infection, resulting in widespread inflammation and multiple organ failure [9]. The treatment of sepsis, mainly targeting inflammatory mediators, has only limited success [10]. 

Myrrh is an oleo-gum resin obtained from *Commiphora *species plants (Burseraceae). *Commiphora *species plants are grown as the shrub in desert regions, particularly in north east Africa and the Middle East. The use of Myrrh for its antiseptic properties dates back to biblical times. Apparently, because of its antimicrobial activity, Myrrh has historically been used, alone and in combination with other herbal products, to treat infections and inflammations. Previous phytochemical studies of Myrrh have reported the isolation of resin components including *α*-, *β*-, and *γ*-commiphoric acids, and *α*- and *β*-heerabomyrrholic acids [11, 12]. Myrrh has showed the anti-inflammatory effects in carcinoma cells [13, 14]. The protective effects of Myrrh on gastric ulcer was attributed to its effect on mucus production and an increase in nucleic acid and nonprotein sulfhydryl concentrations, which appears to be mediated through its free radical-scavenging, thyroid-stimulating, and prostaglandin-inducing properties [15]. Therefore, in the present study, we investigated anti-inflammatory properties of Myrrh. We evaluated its effects on LPS-induced inflammatory mediators and on the CLP model of sepsis and its possible mechanisms underlying anti-inflammatory activities, including MAPKs and NF-*κ*B.

## 2. Subjects and Methods

### 2.1. Chemicals and Reagents

RPMI-1640, fetal bovine serum (FBS), penicillin, and streptomycin were obtained from Gibco BRL (Grand Island, NY, USA). Enzyme-linked immunosorbant assay (ELISA) antibodies for detection of mouse IL-1*β*, IL-6, and TNF-*α* were purchased from R&D Systems (Minneapolis, MN, USA). LPS from *Escherichia coli* 011:B4 was purchased from Sigma-Aldrich Chemical (St. Louis, MO, USA). Antibodies against total and phospho-specific MAPKs (ERK 1/2, JNK, and p38) and phospho-specific inhibitory kappa-B (I*κ*-B)*α* and JNK inhibitor, SP600125 were from Cell Signaling Technology (Beverly, MA, USA). I*κ*-B*α* monoclonal antibody and peroxidase-conjugated secondary antibody were purchased from Santa Cruz Biotechnology, Inc. (Santa Cruz, CA, USA). Prestained sodium dodecyl sulfate-polyacrylamide gel electrophoresis markers were from Bio-Rad (Hercules, CA, USA). Trizol reagent and polymerase chain reaction (PCR) reaction kits were purchased from Invitrogen Corporation (Carlsbad, CA, USA). iNOS, COX-2, TNF-*α*, IL-1*β*, IL-6, and *β*-actin oligonucleotide primers were purchased from Genotech (Daejeon, South Korea). 

### 2.2. Preparation of Myrrh Water Extract

Myrrh was purchased from a standard commercial source (Omni Herb, Daegu, Korea). The herb's identity was confirmed at the Korean drug test laboratory. Voucher specimens were deposited at the College of Oriental Medicine Herbarium of Wonkwang University. The Myrrh (100 g) was prepared by decocting the dried prescription of herbs with boiling distilled water (1 L). The decoction time was approximately 2 h. The water extract was frozen at −80°C, then freeze-dried to be powdered (11.56 g). The yield of extract was 11.56%. The powder was extracted with distilled water and filtered. The filtrates were stored at 4°C until use.

### 2.3. Peritoneal Macrophages Culture

Thioglycollate- (TG-) elicited peritoneal macrophages were harvested 4 d after intraperitoneal injection of 3 mL TG [16]. Peritoneal lavage was performed using 8 mL of RPMI supplemented with 10% heat-inactivated FBS. After incubation for 3 h, nonadherent cells were removed and adherent cells were replaced by new medium, then treated with LPS in the absence or presence of Myrrh.

### 2.4. MTT Assay

Cell viability was assayed using a modification of a colorimetric technique based on the ability of live cells to convert the tetrazolium compound 3-(4,5 dimethylthiazol)-2,5-diphenyl tetrazolium bromide (MTT) into purple formazan crystals. MTT (5 mg/mL) was dissolved in Kreb's-Henseleit buffer (115 mM NaCl, 3.6 mM KCl, 1.3 mM KH_2_PO_4_, 25 mM NaHCOs, 1 M CaCl_2_, and 1 M MgCl_2_). Fifty *μ*L of the mixture was added to each well. After 30 min incubation at 37°C, the suspension was removed and the formazan crystals formed were dissolved in 200 *μ*L of dimethyl sulfoxide. Aliquots from each well were seeded in wells of a 96-well plate in duplicate and assayed at 540 nm using a micro-ELISA plate reader. The number of Myrrh-treated viable cells was expressed as a percentage of the control (the cell without Myrrh) maintained for a comparable time period.

### 2.5. Measurement of NO Concentration

Murine peritoneal macrophages (2 × 10^5^ cells/well) were pretreated with Myrrh for 1 h and then stimulated with LPS (100 ng/mL) for 24 h. To measure the NO concentration 100 *μ*L aliquots were removed from conditioned medium and incubated with an equal volume of Griess reagent (1% sulfanilamide/0.1% N-(1-naphtyl)-ethylenediamine dihydrochloride/2.5% H_3_PO_*4*_) at room temperature for 10 min. The absorbance at 540 nm was then determined.

### 2.6. Western Blot

Murine peritoneal macrophages (5 × 10^6^ cells/dish) were stimulated with 100 ng/mL of LPS. Whole cell lysates were made by boiling peritoneal macrophages in sample buffer (62.5 mM Tris-HCl (pH 6.8), 2% sodium dodecyl sulfate, 20% glycerol, and 10% 2-mercaptoethanol). Proteins in the cell lysates were then separated by 10% SDS-PAGE and transferred to a nitrocellulose membrane. The membrane was then blocked with 5% skim milk in PBS-Tween-20 for 2 h at room temperature and incubated with antibodies to iNOS, COX-2, phosphorylated ERK 1/2, phosphorylated JNK, phosphorylated p38, phosphorylated I*κ*-B*α* and I*κ*-B*α* for overnight. After washing three times, each blot was incubated with secondary antibody for 1 h and the antibody-specific proteins were visualized using an enhanced chemiluminescence detection system (Amersham, Piscataway, NJ, USA) according to the manufacturer's recommended protocol.

### 2.7. PGE_2_ Assay

The PGE_2_ level was measured by immunoassay kits according to the manufacture's protocol (Stressgen Biotechnologies, MI, USA).

### 2.8. ELISA

Murine peritoneal macrophages (1 × 10^6^ cells/well) were stimulated with 100 ng/mL of LPS and/or various concentrations of Myrrh for 24 h. Culture supernatants were collected and stored at −80°C until use. Cytokine levels in the supernatants, blood, and peritoneal fluid were determined using a commercial system (R&D Systems) according to the manufacturer's instructions. The ELISA was devised by coating 96-well plates with specific for TNF-*α*, IL-1*β*, and IL-6. The coated plates were washed with PBS containing 0.05% Tween-20. All reagents used in this assay were incubated for overnight at 4°C. Recombinant TNF-*α*, IL-1*β*, and IL-6 were diluted and used as a standard. Serial dilutions starting at 20 ng/mL were used to establish the standard curve. Assay plates were exposed sequentially to biotinylated mouse TNF-*α*, IL-1*β*, and IL-6 avidin peroxidase, and a substrate solution of 2,2′-azino-bis-[3-ethylbenzthiazoline-6-sulfonic acid] (ABTS) containing 30% hydrogen peroxide. The plates were read at 405 nm.

### 2.9. RNA Quantification

Total cellular and liver RNA was isolated by using the Trizol (Invitrogen, Carlsbad, CA, USA) extraction according to the manufacture's instructions. Total RNA (1 *μ*g) was converted to cDNA by 200 units of reverse transcriptase and 500 ng of oligo-dT primer in 50 mM Tris-HCl (pH 8.3), 75 mM KCl, 3 mM MgCl_2_, 10 mM DTT, and 1 mM dNTPs at 42°C for 1 h. The reaction was stopped by heating at 70°C for 15 min and 3 *μ*L of the cDNA mixture was used for enzymatic amplification. PCR was performed in 50 mM KCl, 10 mM Tris-HCl (pH 8.3), 1.5 mM MgCl_2_, 0.2 mM dNTPs, 2.5 units of Taq DNA polymerase, and 0.1 *μ*M each of primers for iNOS, COX-2, TNF-*α*, IL-1*β*, IL-6, and *β*-actin. Amplifications of conditions were denaturation at 94°C for 3 min for the first cycle and for 45 s starting from the second cycle, annealing for iNOS at 55°C for 40 s, COX-2 at 65°C for 40 s, TNF-*α* at 62°C for 20 s, IL-1*β*, IL-6, *β*-actin at 58°C for 45 s, for 35 cycles. Final extension was performed at 72°C for 7 min. PCR products were electrophorased on a 1.5% agarose gel and stained with ethidium bromide. Primers used were 5′-AGC CCA ACA ATA CAA ATG ACC CTA-3′ (forward) and 5′-TTC CTG TTG TTT CTA TTT CCT TTGT-3′ (reverse) for iNOS, 5′-CAC TCA GTT TGT TGA GTC ATTC-3′ (forward) and 5′-GAT TAG TAC TGT AGG GTT AAT G-3 (reverse) for COX-2, 5′-ATG AGC ACA GAA AGC ATG ATC-3′ (forward) and 5′-TAC AGG CTT GTC ACT CGA ATT-3′ (reverse) for TNF-*α*, 5′-AGT ATC ACT CAT TGT GGC TG-3′ (forward) and 5′-TCA CAG AGG ATG GGC TCT TC-3′ (reverse) for IL-1*β*, 5′-TCA CAG AGG ATG GGC TCT TC-3′ (forward) and 5′-CAT CCA GTT GCC TTC TTG GGA-3′ (reverse) for IL-6, 5′-TGT GAT GGT GGG AAT GGG TCA G-3′ (forward) and 5′-TTT GAT GTC ACG CAC GAT TTC C-3′ (reverse) for *β*-actin.

### 2.10. Nuclear Extraction and NF-*κ*B Binding Activity

Murine peritoneal macrophages were plated in 100-mm dishes (1 × 10^7^ cells/dish). The cells were treated with Myrrh (0.5 mg/mL), stimulated with LPS for the time as indicated. Nuclear extracts were prepared by nuclear extraction kit from Panomics (CA, USA). The isolated cytosol was kept at −80°C for western blot. The nuclear extraction was used for examining NF-*κ*B binding activity. The NF-*κ*B binding activity was measured by NF-*κ*B p65 binding activity assay kit from Active Motif (CA, USA).

### 2.11. Cecal Ligation and Puncture

We performed CLP as described previously [17]. Briefly, we anesthetized the mice and shaved the abdominal wall. After midline laparotomy, we exposed the cecum, ligated below the ileocecal valve without causing intestinal obstruction and then punctured with a 21 G needle. We assessed clinical score every 24 h after CLP, We determined survival rate daily for 8 d after CLP.

### 2.12. Bacterial Counts

We determined bacterial counts as described previously [17]. Briefly, we collected peritoneal lavage fluids using PBS and blood 6 and 24 h after CLP. We serially diluted the samples, plated them on agar dishes and incubated the dishes for 24 h at 37°C. Bacterial counts were determined by counting colony-forming units.

### 2.13. Peritoneal Leukocyte Counts

Peritoneal leukocyte counts were measured by Diff Quik staining kit according to the manufacture's protocol (Sysmex, Japan).

### 2.14. Histopathology Analysis

Liver of mice were excised at 24 h after CLP. The liver samples were fixed in 10% formaldehyde at room temperature. Then the samples were paraffin embedded, cut into 4 *μ*m sections, and stained with hematoxylin-eosin.

### 2.15. Aspartate Aminotransferase (AST)/Alanine Aminotransferase (ALT) Analysis

The fresh serum was used for assaying AST/ALT using biochemical kits (Span Diagnostics, Surat, France).

### 2.16. Myeloperoxidase (MPO) Estimation

Neutrophil sequestration in the liver was quantified by measuring tissue MPO activity. Tissue samples were thawed, homogenized in 20 mM phosphate buffer (pH 7.4), and centrifuged (13,000 rpm, 10 min, 4°C). The pellet was resuspended in 50 mM phosphate buffer (pH 6.0) containing 0.5% hexadecyltrimethylammonium bromide (Sigma-Aldrich). The suspension was subjected to four cycles of freezing and thawing and was further disrupted by sonication for 40 s. The sample was then centrifuged (13,000 rpm, 5 min, 4°C), and the supernatant was used for the MPO assay. The reaction mixture consisted of the supernatant, 1.6 mM tetramethylbenzidine (Sigma-Aldrich), 80 mM sodium phosphate buffer (pH 5.4), and 0.3 mM hydrogen peroxide. This mixture was incubated at 37°C for 110 s and the absorbance was measured at 450 nm.

### 2.17. Statistical Analysis

The results are expressed as the mean ± S.D. of independent experiments. Two-way ANOVA was used to analyze the statistical significance of the results between or among groups, and if statistically significant, *post hoc* analysis using the Duncan method was followed as a multiple comparison among groups. All statistical analyses were performed using SPSS, version 10.0 statistical analysis software. *P < 0.05 were considered to indicate statistical significance.

## 3. Results

### 3.1. Effects of Myrrh on LPS-Induced Production of IL-1*β*, IL-6, and TNF-*α*

**Figure 1:**
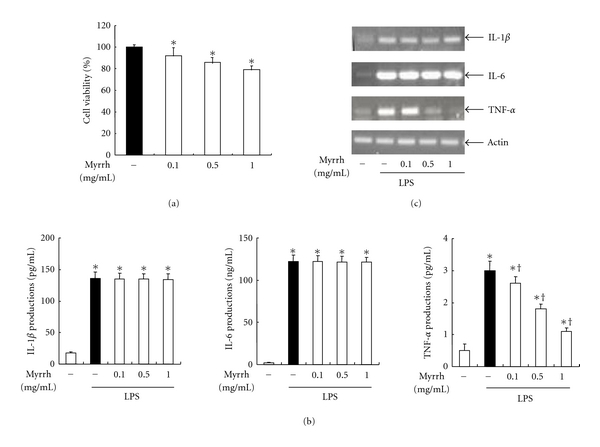
Effects of myrrh on LPS-induced cytokine production. (a) The effects of myrrh on cell viability were measured by MTT assay. Peritoneal macrophages were incubated with or without Myrrh as indicated dose for 24 h. (b) The cells were treated with Myrrh as indicated dose, then stimulated with LPS (100 ng/mL) for 24 h. The supernants were harvested, then the levels of IL-1*β*, IL-6, and TNF-*α* were measured by ELISA as described in Subjects and Methods. (c) The cells were pretreated with myrrh as indicated dose for 1 h, then stimulated with LPS (100 ng/mL) for 24 h. Cells were taken for RT-PCR. Total RNA (1 *μ*g) was prepared for the RT-PCR of IL-1*β*, IL-6, TNF-*α*. The IL-1*β* (126 bp), IL-6 (488 bp), and TNF-*α* (276 bp) were detected by agarose gel electrophoresis, as described in Subjects and Methods. Actin (514 bp) was used as loading control. The values are means ± SD of three independent experiments. *P < 0.05 versus saline. ^+^ *P* < 0.05 versus LPS.

To examine the anti-inflammatory effect of Myrrh on peritoneal macrophages, we analyzed proinflammatory cytokine productions. A prerequisite to study the biological activity of any compound or plant extract is to ensure that they do not have a detrimental effect on cell metabolism. To determine if Myrrh extract affects cell viability, peritoneal macrophages were incubated for 24 h with the extract as indicated dose, and cell viability was evaluated by MTT assay. Myrrh extract did not affect cell death (Figure 1(a)). Thus, a concentration range of 0.1–1 mg/mL was chosen for treatment in the follow-up experiments. We examined the effects of Myrrh on LPS-induced IL-1*β*, IL-6, and TNF-*α* production using ELISA and RT-PCR. We found that the pretreatment of Myrrh extract inhibited LPS-induced production of TNF-*α* but not of IL-1*β* and IL-6 (Figures 1(b) and 1(c)).

### 3.2. Effects of Myrrh on LPS-Induced PGE_2_ and NO Production

**Figure 2:**
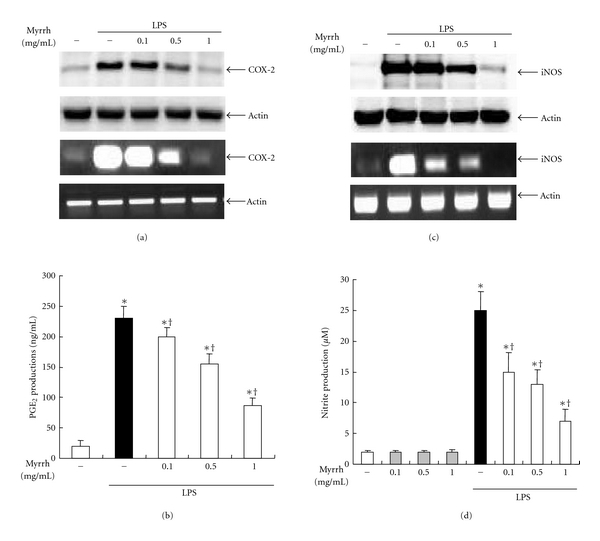
Effects of Myrrh on LPS-induced inflammatory mediators. Peritoneal macrophages were pretreated with myrrh as indicated dose for 1 h, then stimulated with LPS (100 ng/mL) for 24 h. (a) The effects of myrrh on LPS-induced COX-2 protein and mRNA expression, (b) PGE_2_ production, (c) iNOS protein and mRNA expression, and (d) NO production were measured. The cells were taken for western blot and RT-PCR. For western blot, total cellular proteins (20 *μ*g) were resolved by SDS-PAGE, transferred to PVDF membrane, and detected with COX-2 (70 kDa) and iNOS (131 kDa) antibody, as described in Subjects and Methods. Actin (42 kDa) was used as loading control. A representative western blot of three experiments is shown. For RT-PCR, Total RNA (1 *μ*g) was prepared for the RT-PCR of COX-2 and iNOS. The COX-2 (696 bp) and iNOS (734 bp) were detected by agarose gel electrophoresis, as described in Subjects and Methods. Actin (514 bp) was used as loading control. The supernants were harvested, then PGE_2_ and NO levels were measured as described in Subjects and Methods. The values are means ± SD of three independent experiments. *P < 0.05 versus saline. ^+^ *P* < 0.05 versus LPS.

Next, we examined COX-2 protein levels and mRNA expressions. Peritoneal macrophages were pretreated with the indicated concentrations of Myrrh extract for 1 h and then stimulated with 100 ng/mL of LPS for 24 h. Pre-treatment of Myrrh extract inhibited LPS-induced levels of inflammatory mediators such as COX-2 and PGE_2_ (Figures 2(a) and 2(b)). Next, we examined NO production in peritoneal macrophages. The pretreatment of Myrrh extract inhibited LPS-induced iNOS mRNA expressions and protein levels in a dose-dependent manner (Figure 2(c)). As shown in Figure 2(d), in agreement with iNOS results, pretreatment of Myrrh extract inhibited LPS-induced NO production in a dose-dependent manner.

### 3.3. Effects of Myrrh on Activation of NF-*κ*B and MAPK

**Figure 3:**
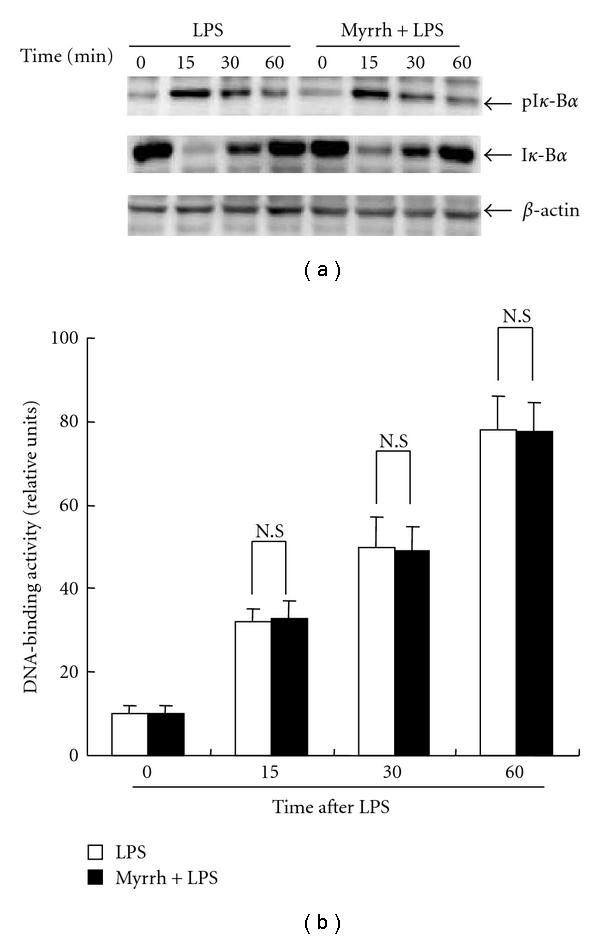
Effects of myrrh on LPS-induced NF-*κ*B activation. (a) Peritoneal macrophages (1 × 10^7^/100 mm dish) were treated with myrrh (0.5 mg/mL) for 1 h, then stimulated with LPS as indicated time points. The cells were harvested for isolation of cytosol and nucleus. The nucleus fraction was performed by Nucleus extraction kit as described in Subjects and Methods. The cytosol was used for western blot. Total cellular proteins (20 *μ*g) were resolved by SDS-PAGE, transferred to PVDF membrane and detected with phosphospefic I*κ*-B*α*  (*36* kDa), anti-I*κ*-B*α*  (*32* kDa) antibody, as described in Subjects and Methods. Actin (42 kDa) was used as loading control. A representative western blot of three experiments is shown. (b) The fractioned nucleus was assayed for DNA (NF-*κ*B p65)-binding activity. The binding activity was measured by NF-*κ*B p65 binding activity kit as described in Subjects and Methods. The values are means ± SD of three independent experiments. *P < 0.05 versus saline.

**Figure 4:**
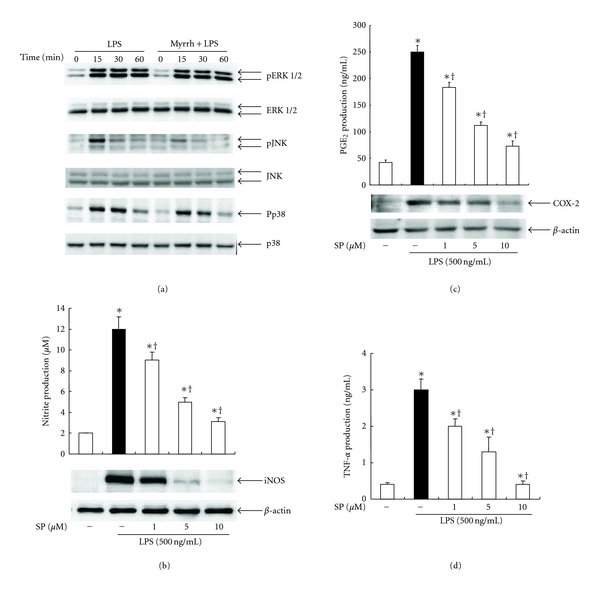
Effects of myrrh on MAPKs activation. (a) Peritoneal macrophages (5 × 10^6^/60 mm dish) were treated with myrrh (0.5 mg/mL) for 1 h, then stimulated with LPS (100 ng/mL) as indicated time points. The cells were harvested for western blot. Total cellular proteins (20 *μ*g) were resolved by SDS-PAGE, transferred to PVDF membrane, and detected with phosphospefic ERK 1/2 (42/44 kDa), JNK (46/54 kDa), and p38 (38 kDa) antibody, as described in Subjects and Methods. ERK, JNK, and p38 were used as loading control. The cells were treated with SP600125, JNK inhibitor as indicated dose for 1 h, then stimulated with LPS for 24 h. (b) The expression of iNOS was detected by Western blot, then nitrite production was measured by Griess method. Actin was used as loading control. (c) The production of PGE_2_ and expression of COX-2 were measured by ELISA and Western blot, respectively. Actin was used as loading control. (d) Production of TNF-*α* was examined by ELISA. Details are described in Subjects and Methods. A representative western blot of three experiments is shown. The values are means ± SD of three independent experiments. *P < 0.05 versus DMSO. ^+^ *P* < 0.05 versus LPS.

To find out inhibitory mechanisms of Myrrh extract on inflammatory mediators, we examined the NF-*κ*B and MAPKs pathway. The cells were treated with Myrrh for 1 h, and then stimulated with LPS as indicated time. As shown in Figure 3(a), Myrrh did not inhibit the phosphorylation and degradation of I*κ*-B*α.* In addition, NF-*κ*B binding activity was also not affected by Myrrh (Figure 3(b)). LPS induced phosphorylation of ERK 1/2, JNK, and p38. However, Myrrh extract inhibited the phosphorylation of JNK but not of ERK 1/2 and p38 (Figure 4(a)). To examine the role of JNK on production of NO, PGE_2_ and proinflammatory cytokines, we used JNK specific inhibitor, SP600125. SP600125 inhibited LPS-induced production of NO, PGE_2_, and TNF-*α* (Figures 4(b), 4(c), and 4(d)).

### 3.4. Effects of Myrrh on CLP-Induced Sepsis

**Figure 5:**
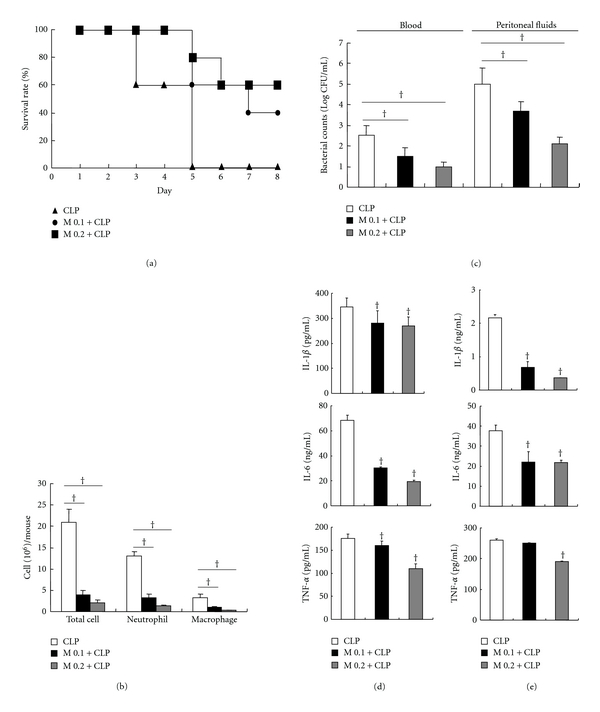
Effects of myrrh on CLP-induced sepsis. Myrrh (0.1 g/kg or 0.2 g/kg) was administrated intraperitoneally 1 h before CLP. The blood and peritoneal fluids were harvested for measuring leukocyte accumulation and bacterial clearance at 24 h after CLP. (a) The survival rate was monitored every day for 8 d, and each group contained 20 mice. (b) Leukocyte accumulation to peritoneal lavage fluids was measured by Diff Quick staining and (c) bacterial clearance was measured in blood and peritoneal lavage fluids. To examine cytokine production, the blood and peritoneal lavage fluids were obtained 6 h after CLP. The cytokine expression in (d) serum and (e) peritoneal lavage fluid were measured by ELISA. The values are means ± SD of three independent experiments. *P < 0.05 versus CLP.

To examine the effect of Myrrh on CLP-induced sepsis, we administrated saline or Myrrh (0.1 g/kg or 0.2 g/kg) intraperitoneally 1 h before CLP. In the sepsis experiment (using 21-gauge needles), by day 8 after CLP, all of the saline treated mice were dead (0 of 20 mice), whereas Myrrh (0.2 g/kg) treated group survived by 60% (12 of 20 mice) (Figure 5(a)). Mice were sacrificed at 6 h or 24 h after CLP; the peritoneal cavity was lavaged and the numbers of inflammatory cells were quantified. The treatment of Myrrh resulted in reduced numbers of neutrophils and macrophages (Figure 5(b)). We investigated whether the increased survival of mice was related to enhance bacterial clearance. To determine the impact of the recruited phagocytes on the numbers of peritoneal and blood bacteria, serial dilutions of peritoneal lavage fluid and blood were cultured on blood agar plates. The bacterial load in blood and peritoneal cavity was determined by CFU count at 24 h after CLP. As shown in Figure 5(c), treatment of mice with Myrrh resulted in increase of bacterial clearance in blood and peritoneal fluids. In our mouse model, cytokines in serum and peritoneal lavage fluid were also reduced at 6 h after CLP in mice treated with Myrrh (Figures 5(d) and 5(e)).

### 3.5. Effects of Myrrh on CLP-Induced Liver Injury

**Figure 6:**
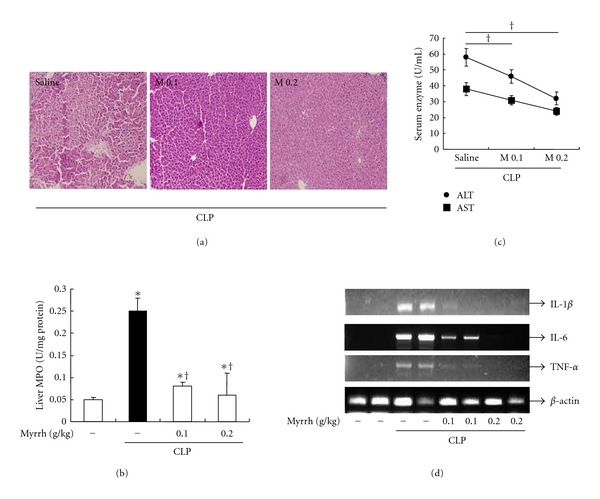
Effects of myrrh on CLP-induced liver damage. Myrrh (0.1 g/kg or 0.2 g/kg) was administrated intraperitoneally 1 h before CLP. (a) Representative H&E-stained sections of liver in mice given saline or myrrh (0.1 or 0.2 mg/kg) 1 h before CLP. 24 h after CLP, mice were sacrificed, then liver and blood were harvested. (b) Liver neutrophil infiltration was assessed by MPO activity. (c) The ALT and AST levels were measured in serum using biochemical kits as described in Subjects and Methods. (d) mRNA levels of cytokines were measured in liver by RT-PCR. The values are means ± SD of three independent experiments. *P < 0.05 versus saline. ^+^ *P* < 0.05 versus LPS.

Next, we examined the effect of Myrrh on liver injury of septic mice. Myrrh treatment remarkably inhibited the inflammatory cells and tissue degeneration in liver tissues (Figure 6(a)). To ascertain the effect of Myrrh on neutrophil infiltration into liver tissues, we examined the MPO activity in tissues of septic mice. Consistent with findings from histopathological examination, MPO activity in liver tissues of septic mice was increased. However, Myrrh treatment resulted in significant reduction of MPO activity live tissues of mice (Figure 6(b)). To further determine the hepatoprotective effect of Myrrh, we examined serum AST and ALT activities in septic mice. As shown in Figure 6(c), CLP resulted in significant increase of AST and ALT activities compared with sham operation. However, treatment of mice with Myrrh reduced AST and ALT activities. Next, we examined the effect of Myrrh on cytokine expressions in liver from CLP-induced septic mice. Myrrh treatment reduced the expressions of IL-1*β*, IL-6, and TNF-*α* mRNA in liver (Figure 6(d)).

## 4. Discussion

Myrrh is the dried oleo-gum resin of a number of *Commiphora* species, and it has been known to contain resin, essential oil, and gum [18]. Many studies investigated the pharmacological effects of Myrrh [19–21]. However, the effects and the mechanisms underlying the protective effects against sepsis and anti-inflammatory activities of Myrrh are poorly defined. In this study, pretreatment of myrrh inhibited LPS-induced production of proinflammatory cytokines, NO, and PGE_2_ in peritoneal macrophages (Figures 3 and 4). We also found that myrrh reduced CLP-induced mortality and inhibited CLP-induced production of IL-1*β*, IL-6, and TNF-*α* in serum and liver (Figures 5 and 6). These results suggest that myrrh may have anti-inflammatory properties.

LPS induces proinflammatory cytokine productions such as TNF-*α*, IL-1*β*, and IL-6. Among these, TNF-*α* is one of the most important cytokines and is required for the synergistic induction of NOS in LPS-stimulated macrophages. TNF-*α* elicits a number of physiological effect, such as septic shock, inflammation, cachexia, and cytotoxicity [22]. In the present study, our results demonstrated that Myrrh (0.1 ~ 1 mg/mL) inhibits LPS-induced production of TNF-*α* but not of IL-1*β* and IL-6 (Figure 1). We further investigated the effect of myrrh (0.1 ~ 1 mg/mL) on NO and PGE_2_ production. NO, generated from L-arginine by iNOS, is an important regulatory molecule in a range of physiological and pathological processes [23]. iNOS produce micromolar range of NO that is responsible for a number of pathological conditions including various inflammatory diseases. Our results demonstrated that Myrrh inhibited the production of NO through suppression of iNOS expression (Figures 2(c) and 2(d)). Another key inflammatory mediator is PGE_2_, which is produced by COX [24]. PGE_2_ is significantly elevated in the plasma of septic or injured patients and is thought to be a component of the resultant immune suppression associated with augmented rates of infection and mortality [25]. COX enzyme has two isoforms of constitutive form (COX-1) and inducible form (COX-2) [24]. The present study demonstrated that myrrh (0.1 ~ 1 mg/mL) inhibited the production of PGE_2_ through the suppression of COX-2 expression (Figures 2(b) and 2(c)). 

To further characterize the inhibitory effect of Myrrh (0.5 mg/mL) on inflammatory response, we examined the effects of myrrh on the activation of transcription factor NF-*κ*B and MAPKs, which regulate the expression of many immune and inflammatory genes. NF-*κ*B is known to play a critical role in the regulation of cell survival genes and to coordinate the expressions of proinflammatory enzymes and cytokines, such as iNOS, COX-2, TNF-*α*, and IL-6 [26]. The activity of NF-*κ*B is regulated by an inhibitory protein, I*κ*-B*α*. When cells are activated, the inhibitory proteins undergo phosphorylation, which subsequently leads to the degradation of I*κ*-B*α*. The loss of I*κ*-B*α* results in the release of free NF-*κ*B units that translocate from the cytoplasm to the nucleus, where they may trigger the transcription of specific target genes. In the present study, we found that Myrrh did not inhibit LPS-induced phosphorylation and degradation of I*κ*-B*α* and NF-*κ*B activity (Figures 4(a) and 4(b)). Our results suggest that inhibitory effects of myrrh on the production of NO, PGE_2_, and TNF-*α* do not involve in NF-*κ*B signaling. 

MAPKs (ERK, JNK, p38) react to diverse extracellular stimuli and regulate various cellular activities including signal transduction and expression of proinflammatory mediators. In this study, we found that Myrrh significantly inhibited activation of JNK but not of p38 and ERK (Figure 5(a)). Using specific JNK inhibitor, SP600125, we demonstrated that LPS-activated macrophages, initiated systemic response which are characterized by the production of NO, PGE_2_, and TNF-*α* through JNK activation. Our results suggested that myrrh might inhibit LPS-induced production of NO, PGE_2_, and TNF-*α* through the inhibition of the JNK pathway (Figures 5(b), 5(c), and 5(d)). 

In the CLP model, bacteria spreading from infection sites and entering the bloodstream are rapidly trapped in the many organs such as liver, lung, kidney, and spleen, bound to the surface of specific target cells and macrophages in the target organ and subsequently killed by infiltrated neutrophils [27, 28]. The organs are impaired in mice with lethal sepsis induced by CLP and also in humans with sepsis, and this impairment is associated with ineffective bacterial clearance, leading to bacterial dissemination and high mortality rates [29]. Several reports have demonstrated that inflammatory cytokines can serve as both makers and mediators of the severity of sepsis and that elevated levels of these cytokines predict mortality following CLP [29–31]. In this experiment, the administration of myrrh (0.1 or 0.2 g/kg) led to a marked increase in survival and an increased bacterial clearance (Figure 5). In contrast with *in vitro* results, myrrh treatment reduced levels of proinflammatory cytokines such as IL-1*β*, IL-6, and TNF-*α* in serum and peritoneal fluids. Therefore, it is likely that Myrrh exerted an anti-inflammatory effect *in vivo* by increasing bacterial clearance and inhibiting proinflammatory cytokines production such as IL-1*β*, IL-6, and TNF-*α*.

The liver plays a central role in the metabolism and supports the defense systems of the body [32]. An impending liver organ dysfunction is therefore an ominous sign in critically ill patients, and the development of hepatic failure during sepsis is associated mortality [33]. Because organ dysfunction is considered to be the end point of damage caused by the excessive inflammatory response of sepsis, we therefore examined the level of AST and ALT, MPO activity, cytokine production, and histology during sepsis. In the present study, we demonstrated that treatment of mice with myrrh reduced plasma AST and ALT level and MPO activity in liver from CLP-induced mice (Figure 6), indicating that myrrh reduced hepatic injury in CLP-induced mice. To explore the mechanisms of the development of sepsis-induced hepatic injury, we investigated whether an excessive hepatic inflammatory response developed sepsis-induced hepatic injury, as evidenced by the fact that a number of inflammatory mediators play pivotal roles in the development of CLP-induced hepatic injury [34, 35]. TNF-*α* and IL-1*β* are the most powerful pathological cytokines causing cell degeneration, apoptosis, and necrosis, and leading to multiple organ dysfunction [36]. In addition, TNF-*α* and IL-1*β* can also boost the other inflammatory mediators [35]. In our study, treatment of myrrh reduced expression of IL-1*β*, IL-6, and TNF-*α* in serum and liver during CLP-induced sepsis (Figures 5 and 6). Histopathological examination, AST/ALT levels, and MPO activity also indicated that myrrh alleviated cellular damage in live tissue. These results supported that the protective effect of myrrh on liver in septic mice might be achieved by inhibition of proinflammatory cytokine overproduction.

In conclusion, we demonstrated that myrrh treatment could show anti-inflammatory effects on LPS-stimulated macrophages and antimicrobial sepsis on CLP model. The pretreatment of myrrh inhibited NO, PGE_2_, and TNF-*α* production through inhibition of JNK pathway. These results could give the conclusion that myrrh has a potential in deactivating inflammatory cells such as macrophages and might be beneficial in inflammatory diseases such as sepsis.

## 5. Authors' Contribution

Both M-S Kim and G-S Bae equally contributed to this work.
